# Experimental Investigation of Mechanical Performance and Gamma Radiation Shielding of Hybrid Magnetite–Dolomite High-Density Concrete

**DOI:** 10.3390/ma19143067

**Published:** 2026-07-16

**Authors:** Muhammad Bilal Waseem, Ahsen Aleem, Muhammad Ihtasham Ali, Asad Naeem, Waqas Rafiq, Riyadh Alturki, Muhammad Imran Khan

**Affiliations:** 1 Civil Engineering Department, Military College of Engineering, National University of Sciences and Technology, Risalpur 24080, Pakistan; mbwaseem.be97mce@student.nust.edu.pk (M.B.W.); aaleem@mce.nust.edu.pk (A.A.); miali.be97mce@student.nust.edu.pk (M.I.A.); asnaeem@mce.nust.edu.pk (A.N.); 2 Department of Civil Engineering, Faculty of Engineering, University of Tabuk, P.O. Box 741, Tabuk 71491, Saudi Arabia; 3 Department of Civil Engineering, College of Engineering, Imam Mohammad Ibn Saud Islamic University (IMSIU), Riyadh 11432, Saudi Arabia; rtalturki@imamu.edu.sa

**Keywords:** heavyweight concrete, hybrid magnetite dolomite aggregates, gamma radiation shielding, cesium-137 (Cs-137), cobalt-60 (Co-60), nuclear power plant shielding

## Abstract

**Highlights:**

- Hybrid magnetite–dolomite concrete outperforms conventional concrete in gamma radiation shielding.
- Mix 1 (50% magnetite) attains 78.78% Cs-137 and 76.86% Co-60 attenuation at 101.6 mm (4 in) thickness.
- The magnetite-forward blend achieves 25.8 MPa compressive strength, exceeding the 20.68 MPa target.
- Dolomite addition enhances ductility and energy absorption with minimal loss in shielding efficiency.
- Mix 1 reduces required wall thickness by 41% compared to the control mix for 99% gamma attenuation.

**Abstract:**

Nuclear infrastructure requires reliable gamma radiation shielding, for which heavyweight concrete offers a practical, structural solution. Conventional concrete provides poor gamma shielding and heat durability, demanding a denser alternative. Prior studies show that magnetite enhances attenuation and strength, while dolomite improves thermal/mechanical stability, yet findings are dispersed across materials and test conditions. Hybrid magnetite–dolomite concrete requires systematic evaluation for simultaneous optimal gamma shielding and mechanical performance under nuclear conditions. Two mixes were produced by partial replacement of coarse aggregate (Mix 1: 50% magnetite, 25% dolomite; Mix 2: 25% magnetite, 50% dolomite), casted and cured per standard practice with compressive strength measured at 7 and 28 days. Gamma attenuation was quantified using Cs-137 and Co-60. Mix 1 achieved 78.78% attenuation for Cs-137 and 76.86% for Co-60, while Mix 2 reached 77.65% and 74.68%, respectively. At 28 days, peak compressive strengths were 25.8 MPa (magnetite), 22.6 MPa (dolomite), and 20.6 MPa (control), with pre-peak energy capacity ranking as follows: magnetite > dolomite > control. Magnetite increased strength and attenuation but sharpened post-peak softening, whereas dolomite enhanced deformability and energy dissipation with minimal loss in shielding. Hybrid concrete satisfied shielding and strength targets and outperformed conventional concrete, with a magnetite-forward blend offering the best overall protection.

## 1. Introduction

The protection of human health from harmful radiation, particularly gamma radiation, has become a critical concern in environments such as nuclear power plants (NPPs), where radiation exposure is inevitable. Today, nuclear power supplies roughly 9% of the world’s electricity through 410–440 operating reactors [1]. Those facilities depend on concrete walls that stop intensely penetrating gamma rays; the denser the concrete, the better the protection and the thinner the wall that is needed. With the global push towards reducing carbon emissions, nuclear energy has emerged as a critical solution due to its ability to produce large amounts of electricity without greenhouse gas emissions. However, the safety of nuclear power plants remains a primary concern, especially in terms of radiation shielding and containment. The increasing safety and operational requirements in nuclear power plants, medical facilities, and radioactive waste repositories have driven significant advancements in radiation-shielding materials. Traditional concrete, while cost-effective and mechanically robust, suffers from degradation under intense gamma radiation and elevated temperatures, prompting the development of heavyweight concrete (HWC) and alternative composites [2].

The most important factor in nuclear power plants is safety, especially when it comes to radiation protection of workers, the nearby population, and nature. Although standard concrete is inexpensive and commonly used, it does not contain the necessary heavy materials that could block gamma radiation [3]. When gamma rays interact with normal concrete, they generate heat causing volumetric expansion and progressive cracking [4]. The severity of these effects depends on radiation intensity and the duration of exposure, and while some progress has been made in understanding these properties, further optimization is essential [5].

Due to the rising demand for enhanced levels of safety and operation of nuclear power stations, hospitals, and radioactive waste sites, there has been immense research into the production of heavyweight concrete (HWC) [6]. The linear attenuation coefficient (μ) and half-value layer (HVL) are the primary parameters used to quantify shielding effectiveness. Research emphasizes that aggregate composition and gradation strongly influence these properties [7,8]. Standard Portland cement concrete poses important restrictions in response to continuous exposure to radiation. Common aggregates like limestone and granite have low attenuation capacity along with poor thermal stability and strength beyond 300 °C [9]. Moreover, gamma-induced alkali–silica reactions in traditional aggregates pose long-term durability threats [10]. WC, defined by a density above 2600 kg/m^3^, has demonstrated substantial improvements in gamma shielding, compressive strength, and neutron attenuation [11]. The use of magnetite results in an enhancement of μ value of up to 37% relative to normal concrete [12] and offers mechanical enhancements such as 21.47% in higher compressive strength [13].

Magnetite (Fe_3_O_4_) is a natural heavy aggregate due to its high density (~5.2 g/cm^3^), iron content, and compatibility with Portland cement systems [14]. Studies confirm magnetite’s superior performance against both gamma and neutron radiation [15]. The joint effect of the increased amount of magnetite and the higher actual bulk density causes improved densification, thus contributing to improved gamma attenuation effectiveness of the concrete mixes. Both of these parameters have a significant impact on improving the gamma radiation shielding efficiency [16]. It has achieved HVL as low as 0.012 cm in mixed radiation shielding settings. At elevated temperatures (~400 °C), magnetite concrete retains significant strength and shielding efficiency, although multiple heating cycles cause progressive degradation in tensile strength and porosity [17]. Still, magnetite remains preferred over barite and hematite due to its cost-effectiveness and ease of sourcing [18]. Dolomite (CaMg(CO_3_)_2_) is a medium-density carbonate mineral with favourable mechanical characteristics, especially under thermal exposure. Despite its lower atomic number compared to magnetite, dolomite exhibits good compressive strength, abrasion resistance, and durability. It has been used in limited studies for radiation shielding [19]. Dolomite is a highly innovative and environmentally friendly material that will not only improve the performance of concrete but will also lead to the use of eco-efficient building techniques [20]. El-Samrah evaluated dolomite concrete and reported adequate gamma attenuation at low-energy photons, alongside compressive strength suitable for structural shielding applications [21]. However, dolomite’s lower attenuation coefficient compared to magnetite limits its standalone application in high-radiation contexts.

Combining different aggregates is a known method of optimizing density, attenuation, and mechanical performance. Prior research has explored magnetite–hematite, barite–limonite, and steel slag–goethite combinations. However, magnetite–dolomite combinations remain understudied despite potential synergy: magnetite contributes density and shielding; dolomite adds thermal stability and reduces cost. This unexplored pairing may offer a balanced performance profile suitable for large-scale nuclear infrastructure [22]. Radiation shielding research has increasingly considered recycled and industrial by-products. Examples include red mud-based synthetic aggregates, steel slag, and siderurgical magnetite. These materials offer both high density and environmental benefits [23]. However, challenges like leachability, variability in composition, and lower thermal endurance limit their universal application. Dolomite, as a natural and abundantly available mineral, presents a low-carbon footprint option compared to synthetic substitutes, especially when combined with high-performance magnetite. Some recent research has also brought to light the need for studying issues of brittleness evolution, damage localization, and instabilities in high-density geomaterials under complicated loading conditions. It is worth pointing out here that factors like the stiffness of materials, their internal energy buildup, and crack development play an important role in determining the post-peak response of materials [24].

The effectiveness of heavyweight concrete depends not only on the aggregates but also on mix design parameters. Studies indicate that low water–cement (w/c) ratios (~0.45–0.50) and optimized cement content improve density and reduce porosity [25,26]. Higher aggregate sizes also correlate with increased attenuation due to decreased interfacial transition zones and scattering paths [25]. Use of supplementary cementitious materials (SCMs) like fly ash and silica fume further improves packing density and long-term durability. Inclusion of supplementary cementing materials in concrete greatly increases its durability in aggressive environments. These materials greatly increase resistance to chloride penetration and sulphate attack because they reduce the amount of calcium hydroxide, increase the binding ability for chlorides, and decrease the availability of reactive aluminates [27]. Radiation shielding concretes are often exposed to cyclic thermal loads, neutron bombardment, and chemical exposure. Durability parameters such as gas permeability, porosity, and microcracking determine long-term performance. Research confirms that concrete with magnetite aggregates exhibits lower gas permeability and better retention of shielding properties after thermal cycling [28]. Dolomite aggregates, known for their thermal resistance, may complement magnetite by limiting thermal-induced expansion or degradation, though this has not been extensively studied.

Table 1 provides a comparative overview of recent experimental studies on hybrid aggregate radiation shielding concrete. The current research literature focuses mainly on the analysis of hybrids like serpentine–magnetite [29], magnetite–barite in self-compacting concrete [30], ilmenite–magnetite [16], and barite GPC [31]. In all these studies, it is observed that hybrid aggregate mixtures perform better than single aggregate concretes in terms of shielding effectiveness along with mechanical properties. However, there is no experimental study on the magnetite–dolomite combination yet. The magnetite–dolomite combination is different from magnetite–barite or ilmenite–magnetite combinations because in the latter two types, both the aggregates provide shielding effectiveness through their high-density and high atomic number properties, but the former one provides shielding effectiveness through magnetite, which is rich in iron content with high atomic number (ρ = 5.17 g/cm^3^), whereas dolomite offers mechanical ductility and thermal stability by its carbonate nature (ρ = 2.87 g/cm^3^) at much lower costs.

Magnetite shows superior gamma shielding performance, durability, and thermal resistance. Dolomite offers mechanical and thermal stability but is rarely used alone due to its limited attenuation properties. Despite extensive work on hybrid aggregates, no systematic study exists on combining magnetite and dolomite, especially examining their synergistic potential across shielding efficiency, mechanical behaviour, and high-temperature endurance. This study aims to fill a vital gap by determining whether this hybrid system can optimize density, improve durability, and balance cost, providing a multifunctional material for next-generation nuclear infrastructure. To bridge this gap, this study has following objectives;

- To develop a high-density concrete using magnetite and dolomite coarse aggregates and establish a mix design achieving superior gamma radiation shielding for nuclear power plant structures.
- To quantify and optimize performance alongside density and workability to identify magnetite and dolomite proportions maximizing strength, long-term stability, and shielding efficiency.

Despite considerable research work on heavy concrete utilizing magnetite, barite, hematite, and hybrid aggregates, few experimental works have been conducted on the utilization of magnetite and dolomite aggregates for the dual purposes of shielding from gamma radiations and improving mechanical properties. The balance between these parameters in hybrid magnetite–dolomite aggregates has not yet been well studied. This study aims to fill this void by conducting experimental investigations into the performance characteristics of the hybrid magnetite–dolomite combination and assessing their engineering and economic feasibility for effective shielding applications at large nuclear facilities.

## 2. Materials and Methods

This section outlines the materials, experimental procedures, and testing methodologies adopted in this research to develop and evaluate high-density concrete for potential use in nuclear power plant applications. The process began with the procurement and characterization of raw materials, followed by the design and optimization of concrete mixes using specialized software. Standardized laboratory procedures were employed for sample casting, curing, and testing to ensure reliability and repeatability of results. Both mechanical and radiation shielding properties were examined through compressive strength testing and gamma ray attenuation experiments using Cesium-137 and Cobalt-60 sources.

### 2.1. Material Procurement

**Figure 1:**
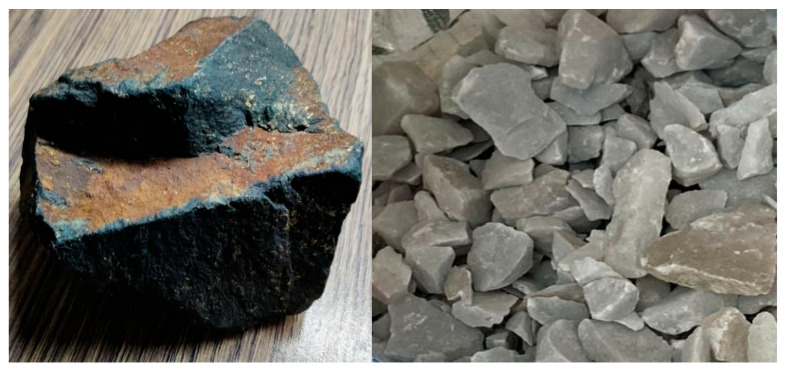
Magnetite (**left**) and dolomite (**right**) aggregates used as coarse aggregate materials in the experimental study.

Magnetite was sourced from the mines located in Taftan, Balochistan, known for their rich mineral deposits. Similarly, dolomite has been procured from the Pakistan Council of Scientific and Industrial Research (PCSIR) in Peshawar as shown in Figure 1.

### 2.2. Concrete Mix Design

The concrete mix was designed in accordance with the guidelines provided by the American Concrete Institute (ACI 211.1) [32]. Prior to mix proportioning, a comprehensive analysis of the physical and chemical properties of both coarse and fine aggregates was performed. This included the determination of maximum aggregate size, specific gravity, fineness modulus, dry rodded unit weight, moisture content, and absorption capacity, following ASTM C33 [33], ASTM C127 [34], and ASTM C128 [35]. The moisture content of the aggregates was carefully considered to ensure accuracy in the final proportions. Table 2 presents the composition of two concrete mix designs, where coarse aggregates are partially replaced with magnetite and dolomite in varying proportions.

The choice of two representative mixes for their performance boundary condition in the hybrid system is made to investigate the trade-off between effectiveness in shielding and ductility in mechanics. The optimum mix should be found by gradient-based optimization in a future study.

The mix design was formulated based on the water-to-cement (W/C) ratio and other essential inputs. The resulting mix proportions were presented in both weight and volume terms. The design aimed to achieve a target strength of 20.68 MPa (3000 psi) with slump range of 76–102 mm (3–4 in). The outputs detailed in Table 3 show the required quantities of water, cement, coarse aggregates, fine aggregates, and air content per cubic yard of concrete, along with the mix proportions by weight and volume. The ratio of the concrete mix was formulated based on proportions of 1:2.3:3.3 (cement–fine aggregate–coarse aggregate) due to the mix design process carried out during the trial mix.

### 2.3. Concrete Testing

In line with the concreting practices commonly followed, a total of three 152 mm × 305 mm (6 × 12 inch) concrete cylinders were prepared as per ASTM C31 [36]. The target compressive strength for this trial mix was established at 20.68 MPa (3000 Psi). To assess the quality and performance of the concrete, standard tests were performed in accordance with their respective ASTM specifications.

**Figure 2:**
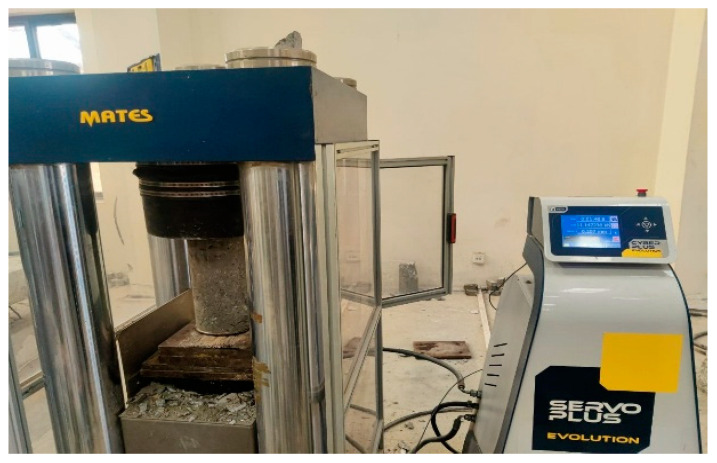
Compressive strength test setup for cylindrical concrete specimen conducted as per ASTM C39 standard using a universal testing machine (UTM).

The compressive strength of the concrete cylinders was evaluated following the procedures specified in ASTM C39 [37], which outlines the standard method for determining the compressive strength of cylindrical concrete specimens (Figure 2). This test provided essential information regarding the load-carrying capacity and overall strength of the concrete.

**Figure 3:**
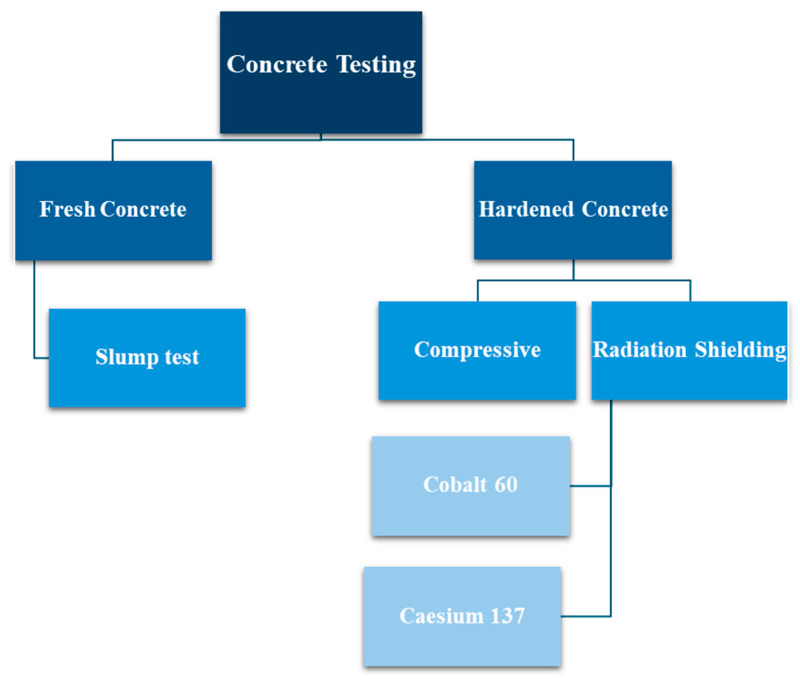
Experimental testing hierarchy outlining the sequence of laboratory procedures and analyses conducted for evaluating the mechanical and radiation shielding performance of concrete specimens.

The following tests were performed at the Military College of Engineering (MCE) and PINSTECH laboratories, as illustrated in Figure 3.

### 2.4. Radiation Shielding Test

**Figure 4:**
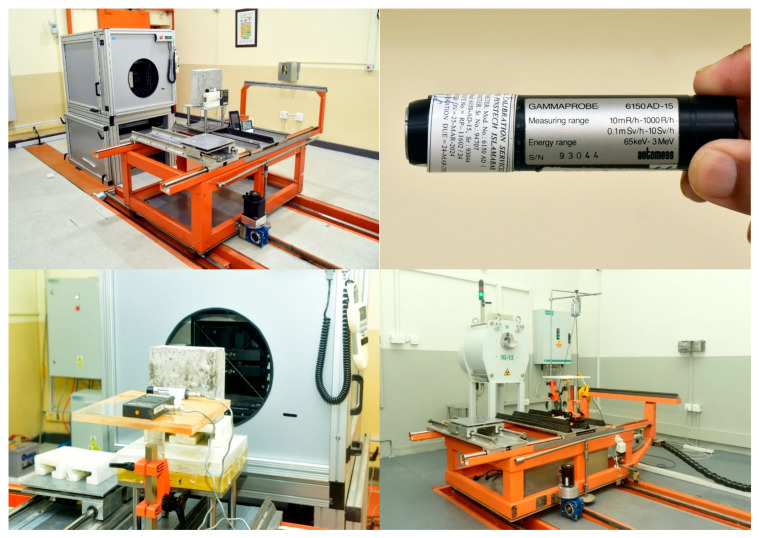
Experimental setup for the gamma radiation shielding test. The concrete specimen was positioned between the radioactive source (Cesium-137/Cobalt-60) and a gamma ray detector to measure transmitted radiation intensity and evaluate shielding effectiveness.

Cesium-137 (Cs-137) and Cobalt-60 (Co-60) radiation sources, with energies of 662 keV and 1200 keV, respectively, were used for radiation experiments at the SSDL, PINSTECH. An AD 6150 radiation detector was used for detecting Cs-137, while a 6150 AD-15 probe in conjunction with 6150 AD-1 detectors was used for Co-60 measurements (Figure 4). The detectors were aligned horizontally with the radiation sources using a laser alignment system. The Cs-137 source was positioned at a fixed distance of 50 cm from the detector, while the Co-60 source was positioned at 208 cm.

A sample thickness of 101.6 mm was chosen based on the technique employed at the PINSTECH Laboratory, Islamabad. In simple terms, the linear attenuation coefficient can be considered as the probability of the interaction of the gamma ray photon with the material per unit length, and it is the property of the material irrespective of the thickness of the sample [38]. The Beer–Lambert law states that μ is mathematically derivable using the value of only one thickness when the intensity of incidence and transmittance is properly measured. In addition, μ is independent of sample shape but dependent on the type of material and radiation energy [39]. This ensures the accuracy and repeatability of the coefficient. The process for each measurement consisted of two parts, taking 20 s in each part: one part with the sample in place and another part with no sample present. This was to achieve statistical significance as well as to reduce any potential errors, and each sample was measured 12 times [17]. This number of repetitions substantially exceeds the 3–5 measurements typically reported in comparable radiation shielding studies [40,41]. For each sample, each measurement of the radiations was performed 12 times to eliminate any randomness in experimental errors. The attenuation figures presented were computed from an average value of several consistent measurements, making the result more reliable. This is a greater number of repetitions compared to most radiation shielding experiments that involve an average of 3–5 measurements.

The consistency of repeated measurements confirmed stable detector response and negligible variation in recorded values.

### 2.5. Sample Casting and Curing

**Figure 5:**
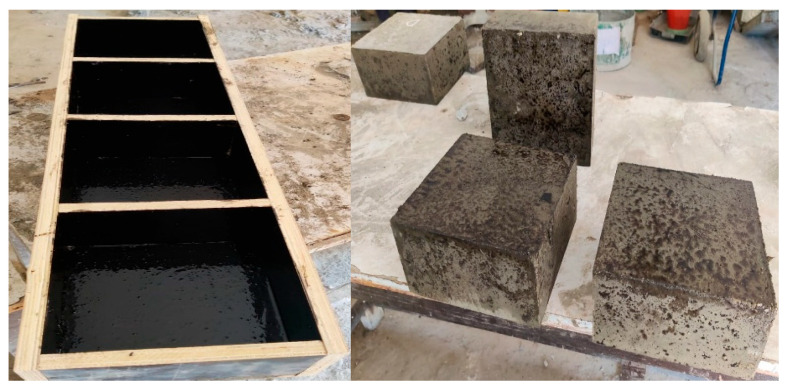
Preparation and casting of concrete shielding blocks using wooden moulds. The produced blocks were employed in radiation attenuation tests against Cesium-137 and Cobalt-60 gamma sources.

In this study, a total of 26 specimens, with 18 specimens for a compressive test of 6 inches in diameter and 12 inches in length, were prepared and tested. For gamma ray testing, specimens with dimensions of 229 mm × 229 mm × 102 mm (9 × 9 × 4 inch) were prepared (Figure 5). This way, four specimens (Cesium and Cobalt) per mix were prepared. A total of 8 specimens against 2 mixes with different percentages of magnetite and dolomite were prepared for gamma ray testing. Before testing, all the specimens for mechanical and attenuation properties were cured in a water tank under controlled laboratory conditions.

### 2.6. Slump Test

The slump test per the American Society for Testing and Materials (ASTM) standard C143 [42] was conducted to evaluate the workability of all three concrete mixes used in this study. A design slump target value of 76.2 to 101.6 mm was determined to guarantee sufficient workability to facilitate placement and consolidation while ensuring mix stability and cohesion.

## 3. Results

### 3.1. Tests Performed on Aggregates

Sieve analysis was conducted to determine the particle size distribution of the coarse aggregates. The maximum aggregate size was found to be 19 mm, indicating a well-graded distribution suitable for dense and uniform concrete. This gradation minimizes voids and segregation, improving compaction and overall strength, which is particularly beneficial for high-density mixes containing magnetite and dolomite.

The three different coarse aggregates used in this study (normal, magnetite, and dolomite aggregates) were treated in the same way as per the ASTM C33 [33], ensuring a consistent and comparable gradation envelope across all mixes. The gradation of the coarse aggregate meeting ASTM C33 [33], Size No. 67 (maximum size of 19 mm), is shown in Table 4. All three coarse aggregates, i.e., normal, magnetite, and dolomite, were made to meet this gradation curve in order to achieve similar packing densities in all mixes. This uniformity in maximum aggregate size eliminates gradation as an independent variable, allowing the observed differences in compressive strength and radiation attenuation between mixes to be attributed directly to aggregate type and density rather than particle size distribution effects.

Specific gravity tests were carried out following ASTM C127 [34] and ASTM C128 [35]. The normal coarse and fine aggregates exhibited values of 2.65 and 2.64, respectively, as presented in Table 5, confirming good quality and low porosity. In comparison, magnetite and dolomite showed higher specific gravities of 4.8 and 2.9, respectively, which significantly enhance the density and radiation-shielding potential of the concrete.

The absorption capacities determined according to ASTM C127 [34] and ASTM C128 [35] were 1.45% for coarse and 3.51% for fine aggregates, as indicated in Table 6. The higher absorption of fine aggregates was considered during mix design to maintain the target water-to-cement ratio. Moisture contents of 1.01% (coarse) and 3.36% (fine) were also recorded to ensure accurate batching adjustments.

**Figure 6:**
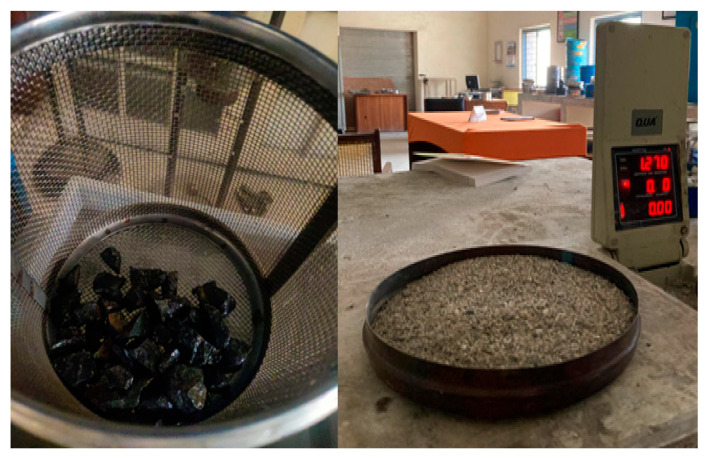
Laboratory testing of aggregates for physical characterization, including specific gravity determination and fineness modulus evaluation as per ASTM standards.

The fineness modulus (FM) of the fine aggregate was determined as 2.34 in accordance with ASTM C136 [43] as shown in Figure 6. This value indicates that the sand used was relatively fine, falling near the lower limit of the standard range, as indicated in Table 6.

### 3.2. Slump Test Results

The results, as displayed in Table 7, indicate that the control mix had an average slump of 91.4 mm, Mix 1 (with 50% magnetite, 25% dolomite, and 25% normal coarse aggregate) had an average slump of 86.4 mm, and Mix 2 (with 25% magnetite, 50% dolomite, and 25% normal coarse aggregate) had a slump of 81.3 mm, all of which met the target slump values. This slight difference between control mix and Mix 2 could be attributed to the shape characteristics of particles used in Mix 2; the aggregates were more angular and had greater surface area as compared to normal coarse aggregate.

### 3.3. Compressive Strength Results

Compressive strength data were collected from the testing and are shown in Table 8. Mix 1 (50% magnetite, 25% dolomite) had a strength value of 25.83 MPa (3747 psi) at day 28, which was 25.2% greater than the strength of the control 20.62 MPa (2991 psi), whereas Mix 2 (25% magnetite, 50% dolomite) produced a strength value of 22.92 MPa (3324 psi), which is 11.1% better than the control mix, at 20.62 MPa (2991 psi). The increasing values of strength as a function of increasing magnetite were because of its angular form with a high hardness (density ρ = 5.17 g/cm^3^). Both hybrid mixes exceeded the designed strength target value of 20.68 MPa (3000 psi) at day 28.

**Figure 7:**
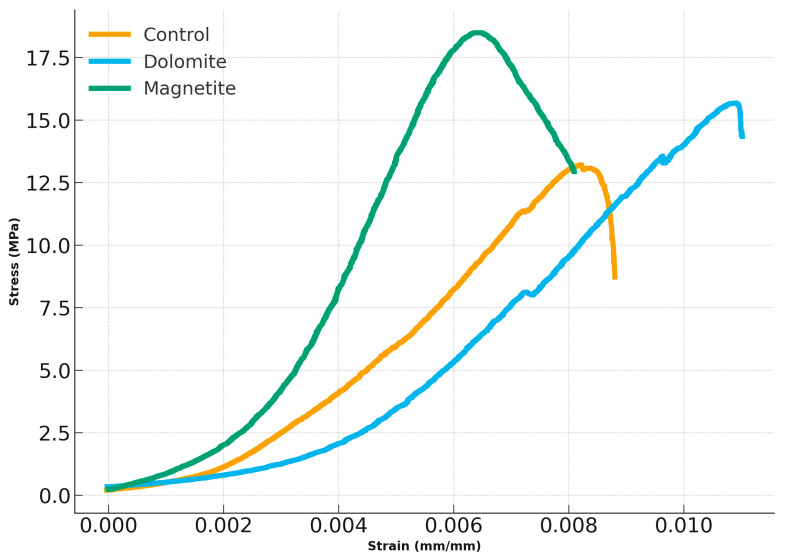
Stress–strain behaviour of control, Mix 1, and Mix 2 concrete specimens at 7 days of curing, showing the comparative development of compressive strength and ductility among the mixes.

At 7 days, all three mixes exhibited the classic compressive response of concrete (Figure 7): an initial “toe” region with low stiffness, as seating effects and micro-void closure dominate; a quasi-linear hardening segment as the matrix and aggregates share load; a nonlinear pre-peak regime as microcracks nucleate and coalesce primarily at the interfacial transition zone (ITZ); a peak stress; and a post-peak softening governed by cracking localization and aggregate/paste failure. The ordering of peak strengths is clear; magnetite is approximately 17.5 MPa at 0.0064 strain > dolomite is approximately 15.4 MPa at ~0.0109 > and the control mix is 13.MPa at ~0.0082. This ranking aligns with expectations from aggregate physics, as magnetite aggregate is denser, stiffer, and typically enhances the load-bearing skeleton earlier in the loading history, while the conventional control mix relies more on paste contribution and a weaker ITZ. Dolomite sits between the two, with better deformability but not the same early stiffness as magnetite.

**Figure 8:**
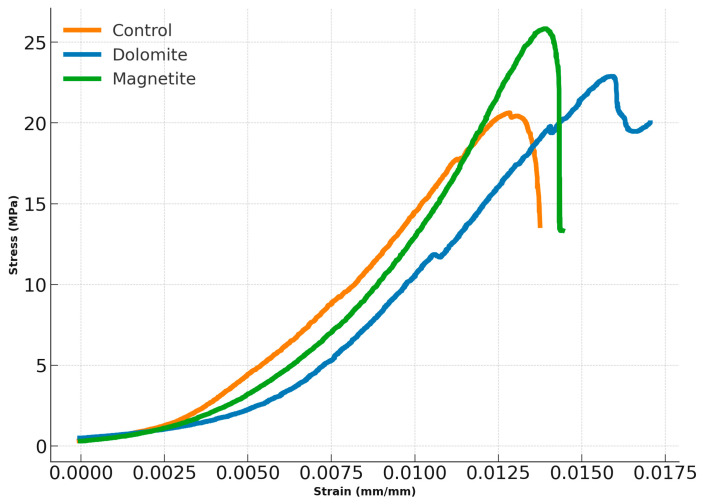
Stress–strain behaviour of control, Mix 1, and Mix 2 concrete specimens at 28 days of curing, showing the comparative development of compressive strength and ductility among the mixes.

The most striking feature is the pace of the magnetite mix picking up stress. By ~0.005 strain, the magnetite curve is already around 14 MPa, whereas control and dolomite are only 6 MPa and 3 MPa, respectively, as illustrated in Figure 8. Mechanically, this implies the effective stiffness is much higher for the magnetite mix once the toe-in is cleared and the load path quickly engages a stiff aggregate skeleton, meaning the paste and ITZ are sufficiently sound at 7 days to transfer load efficiently into the dense magnetite particles. In short, magnetite “locks up” early, reducing compliance and elevating stress for a given strain.

However, there is a trade-off that shows up in the strain at peak. The magnetite mix peaks at 0.006 to 0.0065, notably earlier than dolomite (0.0109) and control (0.0082). This is the signature of a more brittle response. The likely mechanism is modulus mismatch at the ITZ, where the very stiff magnetite particles concentrate stresses in the surrounding paste; as loading proceeds, microcracks initiate and link up along the ITZs, so once the network percolates, the specimen reaches peak and the drop beyond is relatively abrupt.

The dolomite mix climbs more leisurely. At 0.005 strain, dolomite is only 3.5 MPa, but it keeps hardening steadily up to 15.7 MPa at 0.0109. This curve shape shows aggregate stiffness is lower and the ITZ is comparatively more forgiving; microcracks nucleate later and accumulate more gradually, and the system absorbs more inelastic deformation before peak. Up to the peak, the area under the curve comes out largest for dolomite among the 7-day mixes (0.0627 MPa strain to peak vs. 0.0445 for magnetite and 0.0424 for control), indicating greater energy absorption prior to failure. The physical picture is that the lower contrast in stiffness between dolomite and paste reduces stress concentration at the ITZ, delaying unstable crack growth.

The control mix sits between these two, being more compliant than magnetite and less ductile than dolomite. It peaks at 13.2 MPa around 0.0082 strain, with a moderate drop thereafter. This behaviour is consistent with a typical crushed-rock aggregate concrete at an early age.

In all three curves, as strain moves past 0.003 to 0.004, the slope decreases noticeably. This is the regime in which distributed microcracking begins to dominate. In magnetite concrete, because the aggregate carries load efficiently, the transition is sharp: once the ITZ starts to microcrack, the load transfer path degrades quickly, and peak arrives earlier. In dolomite, microcracking is more diffuse and progresses over a wider strain band, so it has a smoother approach to peak and more gradual post-peak softening.

Compressive strength depends on the stiffness and strength of paste (C-S-H gel, capillary porosity), aggregate stiffness and shape, ITZ thickness/defects, and confinement from lateral restraint created by internal aggregate interlock. Magnetite’s high particle stiffness and likely angularity promote early confinement and high apparent stiffness, but a stiffer skeleton also means less tolerance for strain. Dolomite’s more compliant skeleton allows microcracks to spread and blunt, so it can carry load at higher strains, even if ultimate stress is lower. The control mix mirrors the classic concrete response without the extreme effects seen in the other two.

At 28 days, the microstructure matures, more hydration products fill capillary pores, ITZ porosity drops, and paste stiffness increases (Figure 8). All three mixes shift upward and to the right higher strengths and larger strains at peak. Measured peaks move to 25.8 MPa at 0.0139 (Magnetite), 22.6 MPa at 0.0156 (Dolomite), and 20.6 MPa at 0.0128 (Control), as illustrated in Figure 8. Relative to 7 days, this is roughly a +40% (Magnetite), +45% (Dolomite), and +56% (Control) gain in peak stress, with similarly notable increases in the strain at peak. This is a demonstration of ITZ strengthening and paste densification with curing.

Looking closely at the first 1% strain, the ordering there is subtle. In the curve, at 0.010 strain, the control mix is 14.5 MPa, slightly above dolomite (10.6 MPa) and close to magnetite (13.0 MPa). This means at service-level strains, the control mix’s paste and aggregate system is stiffened substantially by curing, closing the gap with magnetite and surpassing dolomite in the mid-strain regime. Dolomite’s lower stiffness means less stress concentration at the ITZ. The curve therefore bends up more strongly in the 0.010–0.015 strain band, catching and then nearing magnetite in stress just before magnetite peaks. This is because dolomite stores more deformation before hitting its maximum stress. The post-peak shapes separate the mixes. Magnetite shows a steep drop after peak, characteristic of a brittle transition where the ITZ/paste around very stiff particles gives way and localized crushing propagates quickly. Dolomite, in contrast, keeps carrying high stress even past 0.017 strain, with only gentle undulations consistent with progressive microcracking, frictional sliding, and aggregate interlock rather than a single catastrophic crushing front. The control mix sits in between a distinct drop after 0.0128 peak but not as abrupt as magnetite.

The area under the curve to peak confirms this maturation. By 28 days, all three increase markedly: the control mix shows a 0.104 MPa–strain ratio, dolomite 0.128, and magnetite 0.119. This ranking means that while magnetite wins on ultimate strength, dolomite wins on pre-peak energy capacity, and the control mix—though it gains the most strength percentage-wise—still exhibits the least energy absorption to peak among the three. This implies that if ductility/energy dissipation is important, dolomite aggregate is attractive; if peak strength and density matter most, magnetite is compelling; and if economy and predictable moderate behaviour are the priority, the control mix is acceptable.

Continued hydration produces more C-S-H and fills capillary voids, improving the stiffness and strength of the paste and ITZ. This why the control mix, which relies more on paste quality than “super-stiff” aggregate, shows the largest percent gain in peak stress (≈+56%). The stiffer aggregates (magnetite) already provided a strong skeleton at 7 days; their relative gain is a bit smaller because the paste is no longer the bottleneck to the same extent.

As the ITZ densifies, the effective contact area and bond between paste and aggregate increase, raising the stresses that can be transmitted before microcracks localize. This particularly helps mixes where the aggregate/paste stiffness mismatch is high (magnetite), evidenced by the much larger strain at peak at 28 days (0.0139 vs. 0.0064 at 7 days). Essentially, the skeleton carries more load for longer before brittle localization kicks in.

The abrupt drop in the magnetite curve after the peak indicates crack localization and crushing of a narrow zone (a “shear band”) that forms once the ITZ gives way. Dolomite’s extended tail suggests distributed damage, microcracks continue to form and slide, but aggregate interlock and frictional bridging maintain load over a wider strain window. The control mix shows a moderate post-peak descent, consistent with a typical aggregate/paste balance. Similar instability phenomena in terms of localized energy dissipation and strain concentration have also been found to exist in the case of studies conducted on brittle geomaterials. Earlier studies have revealed that when the strain energy stored within the structure exceeds a certain limit, there is the possibility of local crack growth and degradation of the structural material causing sudden softening after peak loading. Hence, the observed decrease in stress in the case of magnetite-filled mixes could be due to localized energy dissipation and crack formation [24].

In sum, the 28-day curves demonstrate the expected strengthening and “stretching” of the response with curing, while preserving the aggregate-driven mechanical response. Magnetite provides the highest strength and a sharper post-peak; dolomite demonstrates the highest energy capacity and ductility, while the control mix has solid all-round performance with big curing gains.

### 3.4. Density Comparison

The density comparison among normal, dolomite, and magnetite aggregates is summarized in Table 9. The normal-weight aggregate showed an average density of 2.2 g/cm^3^, while dolomite exhibited 2.87 g/cm^3^ and magnetite 5.17 g/cm^3^. This substantial increase in density with magnetite confirms its effectiveness as a high-density aggregate, directly contributing to the enhanced unit weight and improved radiation attenuation capability of the developed concrete mixes. The replacement of normal aggregates with magnetite and dolomite thus resulted in a significant improvement in density, which directly correlates with the higher compressive strength observed in Mix 1 and Mix 2. The denser microstructure provided better packing, reduced porosity, and improved stress distribution within the concrete matrix.

The increase in weight per unit from the normal aggregate weight of 2.20 g/cm^3^ to 2.87 g/cm^3^ of dolomite and 5.17 g/cm^3^ for magnetite is an indication that the packing density in the concrete is improved by this increasing trend. The increased packing density in Mix 1 due to the larger amount of magnetite in Mix 1 reduces the space available for photons to pass in between aggregates, thus promoting the transfer of loads efficiently between aggregates and paste and thereby improving the compressive strength and linear attenuation coefficient of Mix 1 compared to Mix 2 and the control.

### 3.5. Radiation Shielding Results

The radiation shielding performance of the developed concrete mixes was evaluated using two gamma-emitting isotopes: Caesium-137 (Cs-137) and Cobalt-60 (Co-60). The experimental testing was conducted in a certified laboratory facility, where each concrete specimen was subjected to gamma radiation exposure under controlled conditions, with test results shown in Supplementary Materials.

To achieve statistical consistency, each mixture was tested through 12 repetitions, which is considerably higher than the number of repetitions (3–5) that is commonly used in related studies on shielding effectiveness [40,41]. The average attenuation percentage, SD and CV% for each mixture were determined using 12 repetitions for Cs-137 and Co-60 sources, as indicated in Table 10. The CV% of all the samples was found to be less than 0.5%, thus establishing a high level of repeatability and the absence of any error in testing performed in a certified PINSTECH laboratory.

#### 3.5.1. Performance Against Cesium 137

The results obtained for Cs-137 (energy level ≈ 0.662 MeV) show that both mixes provided significant gamma radiation attenuation, as presented in Table 11. Mix 1, containing a higher proportion of magnetite, achieved the highest attenuation of 78.78%, corresponding to an average linear attenuation coefficient of 0.15 cm^−1^. Mix 2, with a greater dolomite content, exhibited slightly lower attenuation (77.65%) but maintained the same coefficient value, indicating comparable shielding behaviour.

This enhanced shielding ability can be attributed to the high atomic number (Fe = 26) and density of magnetite, which increases the probability of photon interaction through photoelectric absorption and Compton scattering mechanisms. Dolomite, although lower in atomic number, contributes to matrix compactness and uniform distribution of dense aggregates, reducing internal voids and improving overall attenuation efficiency.

The difference between Mix 1 and Mix 2 was marginal (1.4%), suggesting that while magnetite has a stronger influence on radiation resistance, dolomite also plays a constructive role in the formation of a dense, homogeneous structure that supports effective shielding.

#### 3.5.2. Performance Against Cobalt 60

For Co-60 (energy levels ≈ 1.17 MeV and 1.33 MeV), both mixes again demonstrated substantial radiation attenuation, though the overall efficiency was slightly lower compared to Cs-137 due to the higher photon energy, which results in greater penetration. Mix 1 recorded an attenuation of 76.86%, while Mix 2 achieved 74.68%, with attenuation coefficients of 0.144 cm^−1^ and 0.135 cm^−1^, respectively, as shown in Table 12.

**Figure 9:**
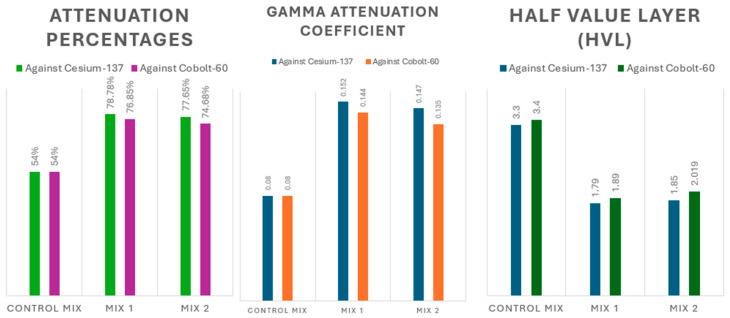
Comparative analysis of radiation shielding performance of control, Mix 1, and Mix 2 concrete specimens under Cesium-137 and Cobalt-60 gamma radiation.

The reduction in attenuation for Co-60 relative to Cs-137 aligns with theoretical expectations, as higher-energy photons exhibit reduced probability of interaction with matter (Figure 9). Nevertheless, the high-density aggregates used in both mixes provided a significant degree of protection even at these energy levels.

The consistently higher attenuation of Mix 1 across both isotopes confirms that magnetite is more effective in gamma shielding due to its high iron content and mass attenuation coefficient. Dolomite’s contribution remains vital, enhancing mechanical strength and aggregate interlocking without significantly compromising radiation performance.

### 3.6. Comparison of Control Mix, Mix 1, and Mix 2 in Terms of Radiation Shielding Performance

Figure 9 illustrates a comparative analysis of the attenuation percentage, gamma attenuation coefficient, and half-value layer for three different concrete mixes—the control mix, Mix 1, and Mix 2—when exposed to gamma radiation from Cesium-137 (0.6 MeV) and Cobalt-60 (1.2 MeV).

According to the literature, the control mix made with conventional aggregates provides all the necessary structural properties; however, it shows low radiation attenuation capability, offering only 54% shielding efficiency against gamma radiation. The corresponding half-value layers derived from the measured coefficients equal 8.38 cm against Cs-137 and 8.64 cm against Co-60. Research also indicates that a total concrete thickness of about 50.8 cm is required for the control mix to block 99% of gamma radiation [3,21,44,45].

In comparison, Mix 1, which incorporates 50% magnetite and 25% dolomite as partial coarse aggregate replacements, shows a substantial improvement with 78.78% attenuation against Cesium-137 and 76.85% against Cobalt-60. The corresponding half-value layers derived from the measured coefficients equal 1.79 in against Cs-137 and 1.89 in against Co-60. This research indicates that a thickness of about 300 mm is required for Mix 1 to block 99% of gamma radiation which is 26% less than control mix.

Mix 2, formulated with 25% magnetite and 50% dolomite, also delivers enhanced performance relative to the control mix, achieving 77.65% attenuation for Cesium-137 and 74.68% for Cobalt-60. The corresponding half-value layers derived from the measured coefficients equal 1.85 in against Cs-137 and 2.019 in against Co-60.

Mix 1 reduces required wall thickness by 41% compared to conventional concrete for 99% gamma attenuation, from 508 mm to 300 mm., representing a 41% decrease in concrete material requirements. The shielding potential of mixing high-density aggregates in different proportions is demonstrated by Mix 2, which performs noticeably better than the control mix despite being marginally less effective than Mix 1. This comparison emphasizes how important aggregate proportioning and selection are to concrete optimization for radiation shielding applications, especially for nuclear infrastructure.

## 4. Discussion

The compressive strength results from present study show a clear influence of aggregate type, with magnetite concrete consistently outperforming dolomite and control mixes at both 7 and 28 days. This aligns with findings in the literature that high-density stiff aggregates such as magnetite significantly enhance compressive strength due to better load distribution and matrix confinement. The results show that dolomite, though less strong than magnetite, still provides a noticeable improvement over standard aggregates due to its higher stiffness and mineral composition.

An important point to consider while designing shielding concrete is whether the compressive strength obtained is sufficient for the required structural purpose. According to ACI 349-13 [46] which is known as “Code Requirements for Nuclear Safety-Related Concrete Structures,” the compressive strength of concrete structures should not be below 3000 psi (20.68 MPa). Both Mixes 1 and 2, having compressive strengths of 25.8 MPa (3747 psi) and 22.6 MPa (3324 psi), respectively, surpass the minimum requirement of 20.68 MPa (3000 psi).

There are three reasons for the cost of magnetite. First, magnetite can be found locally at Taftan, Balochistan, without having to import materials such as barite and ilmenite which would otherwise be necessary in other similar international shield systems. Secondly, the required thickness for 99% gamma shielding is lowered from 508 mm to 300 mm, thus saving on concrete, form work, weight, and time costs to compensate for the increased cost of magnetite per unit volume. Thirdly, substituting magnetite with dolomite for 50% of magnetite material saves money with an efficiency rate of 98.6%.

Synergy in the behaviour of magnetite and dolomite mixtures may be explained using a comparison of performance parameters. Even though Mix 1 with a higher proportion of magnetite recorded the highest attenuation at 78.78%, Mix 2, containing a higher proportion of dolomite, exhibited greater strain tolerance and pre-peak energy absorption at 0.128 MPa–strain compared to 0.119 MPa–strain. This shows that dolomite plays an important role in imparting ductility without sacrificing much shielding efficiency (only about 1.4%). The hybrid mixture records a 46% increase in shielding compared to normal concrete (54% attenuation).

In terms of stress–strain behaviour and failure mode, our findings indicate that magnetite concrete is stiffer but more brittle, failing at lower strains, while dolomite concrete exhibits greater deformability and toughness. These trends are widely supported in the literature. On the other hand, dolomitic and limestone aggregates contribute to a more ductile failure pattern due to improved energy dissipation and crack distribution. The brittleness seen during post-peak in magnetite-containing concrete agrees with the latest literature concerning brittle behaviour and instability in geomaterials under severe loading. Earlier studies conducted concerning brittleness evolution and crack localization have established that stiff and dense materials are prone to experience cracking sooner and display steeper post-peak decline in stresses. This trend could be attributed to the same mechanism for the softening behaviour seen in magnetite-containing concrete in the current study [47].

Dolomite concrete absorbing the most energy prior to peak stress is also well supported. The literature identifies energy absorption and fracture energy as key differentiators in aggregate performance. Magnetite showed high strength; its lower fracture energy is consistent with expectations for dense, brittle aggregates.

Ductility in dolomite concrete can be attributed to the properties of dolomite (CaMg(CO_3_)_2_) regarding chemical and crystalline structure. While magnetite has an inverse spinel structure with highly covalent–ionic bonds between the metal oxides, making deformation difficult, dolomite exhibits a rhombohedral carbonate structure that has well-defined cleavage planes. This results in the particles fracturing along defined lines without complete failure under compression, thus allowing the aggregate to absorb energy incrementally. Additionally, the fact that the elastic modulus of the dolomite, which is composed of calcium–magnesium carbonate, is smaller than that of the magnetite, leads to a reduced modulus mismatch between the two materials at the aggregate–paste interface transition zone, whereby stress concentrations at the ITZ are reduced, microcrack formation is delayed until greater strains have occurred, and crack growth takes place via a more tortuous route. The presence of dolomite’s carbonate surface chemistry further enhances its ability to bond chemically with the calcium silicate hydrate (C-S-H) gel in the ITZ, making it more durable than oxide-based aggregate types. All these phenomena contribute to the high energy absorption capacity of 0.128 MPa strain of Mix 2, which contained dolomite, at 28 days. These properties also allowed the dolomite-containing mix to continue withstanding load after it reached its peak stress, an important feature of toughness for concrete used in nuclear structures under dynamic and seismic forces.

Considering the implementation aspect, it can be stated that the magnetite–dolomite concrete mixture introduced in this research can be considered a useful shielding material for nuclear facilities’ structures from the point of view of engineering practice. From the construction application point of view, mix 1 (50% magnetite and 25% dolomite) should be used in primary shielding walls and biological shielding with gamma ray attenuation being the key design consideration, whereas Mix 2 (25% magnetite and 50% dolomite) can be more suitable for secondary structure shields and ducts with seismic load considerations. Some key points concerning control of construction are that the water-to-cement ratio is kept constant at 0.58, while the aggregate-to-cement ratio remains constant at 3.3 in order to keep the designed density and workability, vibration must be used during placement in order to avoid formation of voids due to the dense nature of the magnetite aggregate. Moreover, the duration of wet curing for a minimum of 28 days is necessary in order to achieve complete densification of the ITZ and reach compressive strength. Economic viability is also improved by using magnetite and dolomite, as they are natural resources readily available in Pakistan; magnetite is imported from Taftan, Balochistan, while dolomite is mined from PCSIR Peshawar. Both these materials are free from any import costs faced by a similar system relying on barite and ilmenite minerals utilized in international research. The system also minimizes the required thickness for 99% gamma shielding from about 508 mm for normal concrete to about 299.7 mm for Mix 1, which is a 41% decrease in concrete quantity and therefore structural dead weight, formwork expenses, and overall building duration for the concrete shields of nuclear reactors. These economic benefits, along with the reduced unit cost of dolomite compared to high-density minerals alone, make the magnetite–dolomite hybrid material more economical and practical.

Regarding the impact of curing time, this study shows substantial improvements in both strength and strain capacity from 7 to 28 days across all mixes. This is expected and supported by the hydration and interfacial transition zone (ITZ) densification literature. That magnetite concrete’s strain at peak nearly doubles from 7 to 28 days suggests improved bond and reduced early-age cracking, which is well aligned with these conclusions.

Attenuation coefficients for Cs-137 (662 keV) and Co-60 (1173/1332 keV) were recorded, thus presenting a broader scope of shielding properties compared to serpentine–magnetite that has recorded no attenuation for Co-60, and magnetite-barite SCC, on which we did not conduct any attenuation studies, as indicated in Table 1. The Cs-137 μ values of Mix 1 (0.152 cm^−1^) and Mix 2 (0.147 cm^−1^) show 23.2% and 35.3% reductions, respectively, compared to ilmenite–magnetite (0.198 cm^−1^) and barite geopolymer (0.235 cm^−1^), while the Co-60 μ values of Mix 1 (0.144 cm^−1^) and Mix 2 (0.135 cm^−1^) are slightly reduced by 1.4% and 21.5%, respectively, compared to ilmenite–magnetite (0.146 cm^−1^) and barite geopolymer (0.172 cm^−1^). The latter two systems only make use of high-density aggregates that come with considerably higher material costs. However, the slight variance in the attenuation of Co-60 is indicative of the effectiveness of the magnetite–dolomite system in providing near equivalent shielding.

The results of this study are consistent with a substantial body of literature indicating that aggregate type critically governs not just the compressive strength but also the stiffness, toughness, and failure behaviour of concrete. Magnetite enhances strength and stiffness but increases brittleness, while dolomite improves ductility and energy absorption.

Together, these results show a three-factor balancing principle for the operation of the magnetite–dolomite hybrid system, where the effectiveness of shielding, compressive strength, and toughness is maximized through the proper ratio of the aggregates. The magnetite, owing to its density (5.17 g/cm^3^) and iron composition, is the main factor in both shielding and strength behaviour, since it increases the probability of interaction between the photons and the material due to the Compton and photoelectric effect. Nonetheless, this is at the expense of brittleness because the large contrast in stiffness between magnetite aggregates and the cement matrix leads to stress concentration at the interface transition zone, which explains the crack localization and brittle behaviour after reaching peak stress. On the other hand, dolomite exerts an influence through lowering stiffness (ρ = 2.87 g/cm^3^) to minimize the difference in stiffness between the aggregate and matrix, spread micro-cracks uniformly, and prolong coalescence. The trade-off in terms of shielding due to this gain in toughness, however, is rather negligible, with only a 1.13% drop in Cs-137 attenuation going from Mix 1 to Mix 2. It can therefore be said that the use of dolomite in the composite material falls into a performance envelope in which there is a significant gain in toughness but little sacrifice in terms of shielding effectiveness. This performance envelope is dictated by the three interlocking elements of magnetite providing strength and shielding, dolomite mitigating brittleness and dissipating energy, and their blend.

## 5. Conclusions

The development of heavyweight magnetite–dolomite concrete achieved high-efficiency gamma shielding and met mechanical performance targets for nuclear infrastructure. For 4-inch specimens, Mix 1 (50% magnetite, 25% dolomite, 25% normal coarse aggregate) attained 78.78% attenuation against Cs-137 with a measured linear attenuation coefficient of μ = 0.152 cm^−1^ and 76.86% against Co-60 with μ = 0.144 cm^−1^. Mix 2 (25% magnetite, 50% dolomite, 25% normal) provided 77.65% attenuation for Cs-137 with a measured linear attenuation coefficient of μ = 0.147 cm^−1^ and 74.68% attenuation for Co-60 with a measured linear attenuation coefficient of μ = 0.135 cm^−1^. The corresponding half-value layers derived from the measured coefficients equal 1.79 in for Mix 1 and 1.85 in for Mix 2 (Cs-137) and 1.89 in for Mix 1 and 2.019 in for Mix 2 (Co-60). For the control mix, the half-value layer is 3.3 against Cs-137 and 3.4 against Co-60. Aggregate densities of 5.17 g cm^−3^ (magnetite) and 2.87 g cm^−3^ (dolomite), compared with 2.20 g cm^−3^ for normal aggregate, underpinned the enhanced unit weight and shielding response.

Quantified mechanical behaviour satisfied and surpassed the 20.68 MPa (3000 Psi) strength target while distinguishing strength–ductility trade-offs driven by aggregate type. Peak compressive strengths at 7 days reached 17.5 MPa at ε ≈ 0.0064 (magnetite), 15.4 MPa at ε ≈ 0.0109 (dolomite), and 13.0 MPa at ε ≈ 0.0082 (control). At 28 days, the peaks increased to 25.8 MPa at ε ≈ 0.0139 (magnetite), 22.6 MPa at ε ≈ 0.0156 (dolomite), and 20.6 MPa at ε ≈ 0.0128 (control). Pre-peak energy capacity ranked dolomite > magnetite > control at both ages (e.g., 28-day areas to peak: 0.128, 0.119, and 0.104 MPa–strain), indicating superior deformability for dolomite and the highest strength for magnetite. Evidence across shielding and mechanical metrics identifies the magnetite-forward hybrid (Mix 1) as the most shielding-efficient configuration, with increased dolomite fractions conferring additional ductility without large losses in attenuation.

## 6. Future Recommendations

Two mixes of the magnetite-rich (Mix 1: 50/25) and dolomite-rich (Mix 2: 25/50) proportions were tested in this study, providing an initial benchmark for the performance of this new hybrid shielding material. As can be seen, the difference in Cs-137 attenuation between Mix 1 and Mix 2 is just 1.13% points (from 78.78% to 77.65%), although there is a gain in pre-peak energy absorption, reflecting the trade-off properties of the material. Further experiments should involve the gradient tests of different proportions in smaller intervals (for example, from 0% to 75% magnetite at 10% intervals), as well as single dopant control group samples (with magnetite only and dolomite only). Individual gradation curves and sieve analysis data were not provided separately in this study due to the uniform maximum aggregate size of ¾ inch used for all coarse aggregates. It would be desirable to conduct individual gradation analysis of each aggregate type in future research work for more comprehensive evaluation of packing density effects.

Further investigations should carry out gradient radiation tests using different thicknesses (for instance, 51, 102, 152, and 203 mm) to prove through experiments the independent thickness of the linear attenuation coefficient values and thus establish the universal application of the μ values derived from the Beer–Lambert Law.

There are many limitations to the current study. Only two levels of the ratio of magnetite to dolomite were tested, thus restricting accurate determination of the optimum level of this ratio. In addition, radiation attenuation measurements were conducted on only one thickness of 101.6 mm (4 in), and no single aggregate samples comprising only magnetite and only dolomite were used. Moreover, heat resistance, neutron attenuation resistance, and resistance to radiation damage due to repeated irradiation were not assessed.

## Figures and Tables

**Table 1:** Comparative overview of recent experimental studies on hybrid aggregate radiation shielding concrete.

Study	Hybrid Combination	Cs-137 μ (cm^−1^)	Co-60 (cm^−1^)	Sources Tested
[29]	Serpentine + Magnetite	~0.16	Nil	Cs-137, Co-60
[30]	Magnetite + Barite (SCC)	Not tested		Nil
[16]	Ilmenite + Magnetite	~0.198	~0.146	Cs-137, Co-60
[31]	Barite Geopolymer (0–100% BR)	~0.235	~0.172	Cs-137, Co-60

**Table 2:** Mix design proportions for control and modified concrete mixes (Mix 1 and Mix 2) developed for enhanced density and radiation shielding performance.

Design Mix	Control Mix	MIX 1	MIX 2
Coarse Aggregate		50% Magnetite	25% Magnetite
	100%	25% Dolomite	50% Dolomite
	Normal Agg.	25% Normal Agg.	25% Normal Agg.
Fine Aggregate	Sand	Sand	Sand
Binding Material	Cement	Cement	Cement

**Table 3:** Concrete mix design specifications (targeted strength 20.68 MPa).

Description	Value	Unit
Admixture	No Admixture	—
Amount of Water (Volume)	0.2076	m^3^ per m^3^ of concrete
Amount of Water (Weight)	207.5212	kg/m^3^
Amount of Cement (Volume)	0.0942	m^3^ per m^3^ of concrete
Amount of Cement (Weight)	296.6382	kg/m^3^
Amount of Coarse Aggregates (Volume)	0.4028	m^3^ per m^3^ of concrete
Amount of Coarse Aggregates (Weight)	1077.6047	kg/m^3^
Amount of Fine Aggregates (Volume)	0.2812	m^3^ per m^3^ of concrete
Amount of Fine Aggregates (Weight)	767.0358	kg/m^3^
Resultant Air Content	0.02	m^3^ per m^3^ of concrete
Water to Cement Ratio	0.58	—
Targeted Strength	20.68	MPa
Targeted Slump	76–102	mm
Mix Design Ratio (by weight)	1:2.3:3.3	—

**Table 4:** Sieve analysis.

Sieve Size	ASTM C33 Passing Limits (%)	Percent Passing—All Coarse Aggregates
25 mm (1 in)	100	100
19 mm (¾ in)	90–100	95
12.5 mm (½ in)	20–55	42
9.5 mm (⅜ in)	0–15	8
4.75 mm (No. 4)	0–5	2

**Table 5:** Specific gravity results of coarse and fine aggregates, including normal, magnetite, and dolomite aggregates used in the experimental program.

Aggregate Type	Specific Gravity
Normal Aggregate	2.65
Magnetite	4.8
Dolomite	2.9
Fine Aggregate	2.64

**Table 6:** Physical properties of coarse aggregates, including maximum aggregate size, fineness modulus, bulk specific gravity, absorption capacity, and moisture content.

Properties of Aggregate	Standard	Coarse Aggregate	Fine Aggregate
Maximum Aggregate size	N/A	3/4″	N/A
Fineness Modulus	ASTM C136	N/A	2.34
Bulk specific gravity	ASTM C127	2.65	2.64
Absorption Capacity	ASTM C127	1.45	3.51
Moisture Content	ASTM C128	1.01	3.36

**Table 7:** Slump test of concrete mixes.

Mix	Target Slump	Average Measured Slump	Remarks
Control	76–102 mm	91.4 mm	Within target range
Mix 1 (50% Magnetite, 25% Dolomite)	76–102 mm	86.4 mm	Within target range
Mix 2 (25% Magnetite, 50% Dolomite)	76–102 mm	81.3 mm	Within target range

**Table 8:** Compressive strength results.

Mixes	7 Days (MPa)	28 Days (MPa)
Control Mix (Normal)	13.2	20.62
Mix 1 (Magnetite 50% and Dolomite 25%)	18.48	25.83
Mix 2 (Magnetite 25% and Dolomite 50%)	15.67	22.92

**Table 9:** Density comparison of normal, magnetite, and dolomite aggregates used in the concrete mixes.

Aggregate Type	Density
Normal Aggregate	2.2 g/cm^3^
Dolomite Aggregate	2.87 g/cm^3^
Magnetite Aggregate	5.17 g/cm^3^

**Table 10:** Statistical error reporting.

Mix	Source	Mean Attenuation (%)	SD (%)	CV (%)
Control mix	Cs-137	54	±0.23	0.43
	Co-60	54	±0.25	0.46
Mix 1	Cs-137	78.78	±0.31	0.39
Mix 2	Cs-137	77.65	±0.28	0.36
Mix 1	Co-60	76.86	±0.35	0.46
Mix 2	Co-60	74.68	±0.33	0.44

**Table 11:** Radiation shielding test results of concrete mixes against Cesium-137 gamma source, showing attenuation percentage and transmitted intensity for control, Mix 1, and Mix 2.

Mixes	Attenuation (%)	Gamma Attenuation Coefficient
Control Mix	54	0.08
Mix 1 (Magnetite 50% and Dolomite 25%)	78.78	0.152
Mix 2 (Magnetite 25% and Dolomite 50%)	77.65	0.147

**Table 12:** Radiation shielding test results of concrete mixes against Cobalt-60 gamma source, showing attenuation percentage and transmitted intensity for control, Mix 1, and Mix 2.

Mixes	Attenuation (%)	Gamma Attenuation Coefficient
Control Mix	54	0.08
Mix 1 (Magnetite 50% and Dolomite 25%)	76.86	0.144
Mix 2 (Magnetite 25% and Dolomite 50%)	74.68	0.135

## Data Availability

The raw data supporting the conclusions of this article will be made available by the authors on request.

## Supplementary Materials

The following supporting information can be downloaded at: https://www.mdpi.com/article/10.3390/ma19143067/s1.
