# Supplementary material for: Experimental Investigation of Mechanical Performance and Gamma Radiation Shielding of Hybrid Magnetite–Dolomite High-Density Concrete
Source: Materials (Basel). 2026 Jul 16;19(14):3067. doi: 10.3390/ma19143067 (PMC13414378; doi:10.3390/ma19143067)
Supplement: Supplementary file 1 [file materials-19-03067-s001.zip › materials-4316365-supplementary.pdf]

PAKISTAN INSTITUTE OF NUCLEAR SCIENCE & TECHNOLOGY
DIRECTORATE OF SYSTEMS & SERVICES
HEALTH PHYSICS DIVISION
SECONDARY STANDARD DOSIMETRY LABORATORY
ISO 9001:2015 CERTIFIED

MATERIAL ATTENUATION REPORT

REPORT NO. **885(8)/25** DATE OF ISSUE:- **4/3/2025**

ORGANIZATION: **NUST UNIVERSITY, ISLAMABAD**

1. **MEASUREMENT CONDITIONS:**

Beam Quality = Co^{60}
Temperature = 20-25 ^{0}C
Pressure = 945-950 mb
Rel. Humidity = 40-50 %

2. **METHODOLOGY:**

A 6150 AD-15 probe in conjunction with 6150 AD-1 detector was placed at 208 cm from Co-60 source was used to measure reference and attenuated responses in Co-60 reference field. Source to front-sample-face distance was set at 186.5 cm. The square field at front surface of the sample defined an edge length of 9.7 cm.

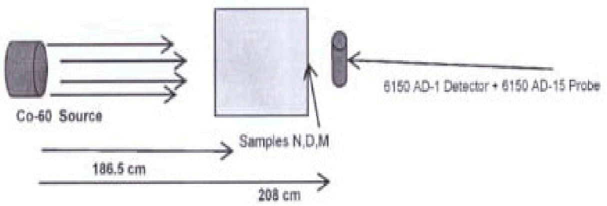

3.

Note : Sample face orientations may vary attenuatiion results if there are material nonuniformities in different layers. Scattering is ignored in the measurements.

Sample Shape = Square-like	Sample ID	Without sample Response (Sv/h)	With Sample Response (Sv/h)	Attenuation (%)
Side length = 22.9 cm, Thickness = 10.2 cm	(Dolomite 50%) D	4.42	1.12	74.68
Side length = 22.9 cm, Thickness = 10.2 cm	(Magnetite 50%) M		1.02	76.85

Report Prepared By | Reviewed by | Head SSDL

PAKISTAN INSTITUTE OF NUCLEAR SCIENCE & TECHNOLOGY
DIRECTORATE OF SYSTEMS & SERVICES
HEALTH PHYSICS DIVISION
SECONDARY STANDARD DOSIMETRY LABORATORY
ISO 9001:2015 CERTIFIED

MATERIAL ATTENUATION REPORT

REPORT NO.: **885(8)/25** DATE OF ISSUE:- **28/02/2025**

ORGANIZATION: **NUST UNIVERSITY, ISLAMABAD**

1. **MEASUREMENT CONDITIONS:**

Beam Quality = Cs^{137}
Temperature = 20-25 ^{0}C
Pressure = 945-950 mb
Rel. Humidity = 40-50 %

2. **METHODOLOGY:**

A AD 6150 radiation detector placed at 50 cm from Cs-137 radiaiton source (IG-13, S 2) was used to measure reference and attenuated responses in Cs-137 reference field. Source to front-sample-face distance was set at 40 cm. The circular field at front surface of the sample defined a diameter of 13 cm.

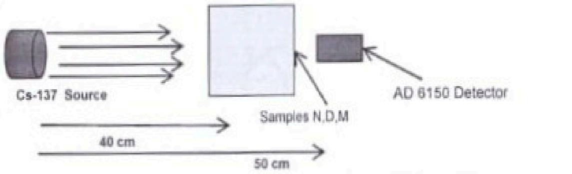

Note : Sample face orientations may vary attenuatiion results if there are material nonuniformities in different layers. Scattering is ignored in the measurements.

3.

Sample Shape = Square-like	Sample ID	Without sample Response (mSv/h)	With Sample Response (mSv/h)	Attenuation (%)
Side length = 22.9 cm, Thickness = 10.2 cm	(Dolomite 50%) D	96.5	21.56	77.65
Side length = 22.9 cm, Thickness = 10.2 cm	(Magnetite 50%) M		20.47	78.78

Report Prepared By   Reviewed by   Head SSDL
